# A feasibility study of ‘The StepSmart Challenge’ to promote physical activity in adolescents

**DOI:** 10.1186/s40814-019-0523-5

**Published:** 2019-11-17

**Authors:** Rekesh Corepal, Paul Best, Roisin O’Neill, Frank Kee, Jennifer Badham, Laura Dunne, Sarah Miller, Paul Connolly, Margaret E. Cupples, Esther M. F. van Sluijs, Mark A. Tully, Ruth F. Hunter

**Affiliations:** 1 UKCRC Centre of Excellence for Public Health/Centre for Public Health, Queen’s University Belfast, Institute of Clinical Science B, Royal Victoria Hospital, Grosvenor Road, Belfast, BT12 6BA Northern Ireland; 2 Centre for Evidence and Social Innovation, School of Social Sciences, Education and Social Work, Queen’s University Belfast, 6 College Park, 69-71 University Street, BT7 1NN Belfast, Northern Ireland; 3 MRC Epidemiology Unit & Centre for Diet and Activity Research (CEDAR), Institute of Metabolic Science, University of Cambridge School of Clinical Medicine, Box 285, Cambridge Biomedical Campus, CB2 0QQ Cambridge, UK; 4 Institute of Mental Health Sciences, School of Health Sciences, Ulster University, Newtownabbey, BT37 0QB Northern Ireland

**Keywords:** Physical activity, Intervention, Behaviour change, Feasibility, Adolescents, Schools, Mixed methods, Randomised controlled trial, Gamification

## Abstract

**Background:**

Inactive lifestyles are becoming the norm and creative approaches to encourage adolescents to be more physically active are needed. Little is known about how gamification techniques can be used in physical activity interventions for young people. Such approaches may stimulate interest and encourage physical activity behaviour. The study investigated the feasibility of implementing and evaluating a physical activity intervention for adolescents which included gamification techniques within schools. We tested recruitment and retention strategies for schools and participants, the use of proposed outcome measures, and explored intervention acceptability.

**Methods:**

This school-based feasibility study of a randomised cluster trial recruited adolescents aged 12–14 years (*n* = 224) from five schools (three intervention; two control) in Belfast, Northern Ireland. The 22-week intervention (The StepSmart Challenge) informed by self-determination theory and incorporating gamification strategies involved a school-based pedometer competition. Outcomes, measured at baseline, and post-intervention (at 22 weeks post-baseline and 52 weeks post-baseline) included daily minutes of moderate to vigorous physical activity (MVPA) (measured using ActiGraph accelerometer), mental wellbeing (Warwick-Edinburgh Mental Wellbeing Scale), social support for physical activity, time preference (for delayed and larger rewards or immediate and smaller rewards), pro-social behaviour (Strengths and Difficulties Questionnaire (SDQ)) and the influence of social networks. The intervention’s acceptability was explored in focus groups.

**Results:**

We invited 14 schools to participate; eight showed interest in participating. We recruited the first five who responded; all five completed the trial. Of the 236 pupils invited, 224 participated (94.9%): 84.8% (190/224) provided valid MVPA (minutes/day) at baseline and 57.2% (123/215) at 52 weeks. All other outcomes were well completed apart from the SDQ (65% at baseline). Qualitative data highlighted that participants and teachers found The StepSmart Challenge to be an acceptable intervention.

**Conclusions:**

The level of interest and high recruitment and retention rates provide support for the feasibility of this trial. The intervention, incorporating gamification strategies and the recruitment methods, using parental opt-out procedures, were acceptable to participants and teachers. Teachers also suggested that the implementation of The StepSmart Challenge could be embedded in a lifelong learning approach to health within the school curriculum. As young people’s lives become more intertwined with technology, the use of innovative gamified interventions could be one approach to engage and motivate health behavioural change in this population.

**Trial registration:**

NCT02455986 (date of registration: 28 May 2015).

## Background

Despite the clear benefits of physical activity, globally 80% of adolescents fall short of meeting required levels [48]. In Northern Ireland, only 14% of 11–16-year-olds are meeting the target of 60 min of daily MVPA [20]. As physical activity patterns track into adulthood [8, 58], adolescence is a crucial period with lifelong effects for health and habits. One suggested explanation is that adolescents are more present-oriented than adults; they overemphasise the ‘costs’ of health-related decisions and discount the value of future benefits [22] and, therefore, can be less likely to engage in health-related behaviours [33, 57]. The use of an engaging intervention using behavioural incentives could help initiate physical activity motivation in more present-orientated individuals by offsetting the immediate ‘costs’ [56].

Gamification is an emerging area in physical activity research that incorporates the use of relevant game design elements in non-gaming contexts [13] which can add fun and enjoyment to physical activity interventions. Gamification theory suggests that it should be possible to turn a routine non-game activity such as travel to school into an engaging and fun game [12]. Although some argue that gamification is just a fad [30, 62], the recent success of Pokémon GO illustrates the potential of gamified interventions for encouraging physical activity [37] and provides insights into how to engage and initiate the most inactive people in physical activity in the short term [63]. Additionally, the recent feasibility testing of gamified interventions using school-based walking competitions to increase active travel in children and adolescents shows promise [7, 23].

Key gamification strategies include using extrinsic (e.g. incentives) and intrinsic (e.g. perceived enjoyment, increasing skills) motivators, the use of challenges and competitions and fostering social connectivity [50]. There is emerging evidence that incentives, which are provided contingent on effort towards the behaviour or successful performance of the target behaviour [38], can be successfully incorporated into physical activity interventions targeting adolescents [10]. Competitions and challenges have shown some success in smoking cessation interventions in adolescents [26] but there is currently limited research investigating the effects of competition on physical activity behaviour change [10]. In addition, friendship networks and the use of peer influencers are associated with physical activity behaviour in adolescents [35, 36, 51, 53] and may influence physical activity behaviour within interventions [24].

Gamification interventions have rarely been grounded in well-established theoretical frameworks [28, 30]. Nonetheless, research has suggested a link between self-determination theory and gamification concepts, as exemplified by the use of extrinsic and intrinsic motivators, competition and challenges and social interaction [47, 52]. Furthermore, the fact that games can make interventions more enjoyable aligns with self-determination theory which postulates that enjoyment is a key aspect of intrinsic motivation [13]. This is reinforced by Best et al. [1] who used survey data to investigate correlates of daily physical activity behaviour in children and adolescents and found fun and enjoyment to be predictors of physical activity.

Although schools appear to be a suitable location in which to deliver physical activity interventions [3, 14, 25], there is a need for more high-quality studies, with process evaluation to understand whether the intervention itself is ineffective or how contextual factors might influence this [31, 39].

This study investigated the feasibility of implementing and evaluating a school-based gamified pedometer competition designed to promote physical activity among 12–14-year-olds, known as ‘The StepSmart Challenge’ (trial number: NCT02455986), which integrates core gamification strategies with self-determination theory. A previous publication details the participants’ experiences of The StepSmart Challenge, in particular the competition formats and use of incentives [9] which appeared to encourage most participants. Participants wore Fitbit Zip pedometers, which appeared to be important for self-directed goal setting, monitoring and immediate feedback.

The specific aims of the feasibility study were to

1. Test recruitment strategies for schools and adolescents;
2. Determine retention rates for schools and adolescents;
3. Assess the appropriateness of proposed outcome measures based on completion rates, missing data and proportion of valid data provided; and
4. Explore the intervention’s acceptability and possible refinements to improve its design.

## Methods

### Study design

The present study was a two-arm parallel feasibility cluster randomised controlled trial comparing ‘The StepSmart Challenge’ and a no-intervention control arm. Five post-primary schools from Belfast, Northern Ireland were recruited. Randomisation took place at the school level, with three schools randomised to the intervention and two schools to the control. The intervention took place from April 2015 to September 2015. Ethical approval was granted by the School of Medicine, Dentistry and Biomedical Science Research Ethics Committee (Queen’s University, Belfast) (Ref: 15.09).

### Recruitment of schools

#### Schools

The sample consisted of post-primary schools in Belfast. For pragmatic reasons, we choose to select our sample from a list of schools which had previously participated in research projects with QUB and were therefore familiar with the time and processes involved. We sent letters of invitation to participate in the trial to a purposive sample of 14 of these schools and made follow-up phone calls to the school principal to explain the purpose of the study. We aimed to invite a mix of schools from affluent and deprived areas (using free school meal eligibility as a proxy measure), and of single-sex and co-educational schools (using public information [15]). Only the first five schools to accept the invitation were recruited due to the scope of the study and the resources available. A stratified randomisation process (stratified by socio-economic status, and whether schools were single-sex or co-educational) was undertaken by an independent statistician to assign schools to the intervention or control group using software available at http://www.randomization.com. As the randomisation was undertaken to assess the feasibility of the randomisation process, all school characteristics were included despite the small number of participating schools. Randomisation resulted in three schools allocated to the intervention group, and two schools to the control group.

#### Participants

Schools were asked to identify ear 9 classes (12–14-year-olds) to participate, aiming to recruit up to 50 adolescents within each school. No formal criteria were applied to class selection; however, we asked teaching staff to recruit classes that were representative of the wider school population in relation to academic ability, gender (if relevant) and perceived physical activity behaviour. We believe the class teachers were well placed to have knowledge regarding perceived physical activity levels, based on, for example, whether adolescents were involved in sports teams or not. Input from teaching staff was important as they were best placed to assess any potential conflicts that may have impacted upon the team component of The StepSmart Challenge.

Following class selection, the research team organised an information session in each school to provide teachers and the selected classes with more details about the study and an opportunity to ask questions. Information sheets, consent forms and parental/guardian opt-out consent forms were provided to adolescents to read and take home to parents/guardians, to allow them time to assimilate the information and make an informed decision. If adolescents wished to take part, they were asked to provide written consent. Adolescents were excluded from the study if (a) their parents informed the school that the adolescent had been advised by their general practitioner not to undertake moderate physical activity, (b) they were not in Year 9, and (c) they had not provided assent or if their parents had completed the parental opt-out written consent form.

### Intervention

**Fig. 1:**
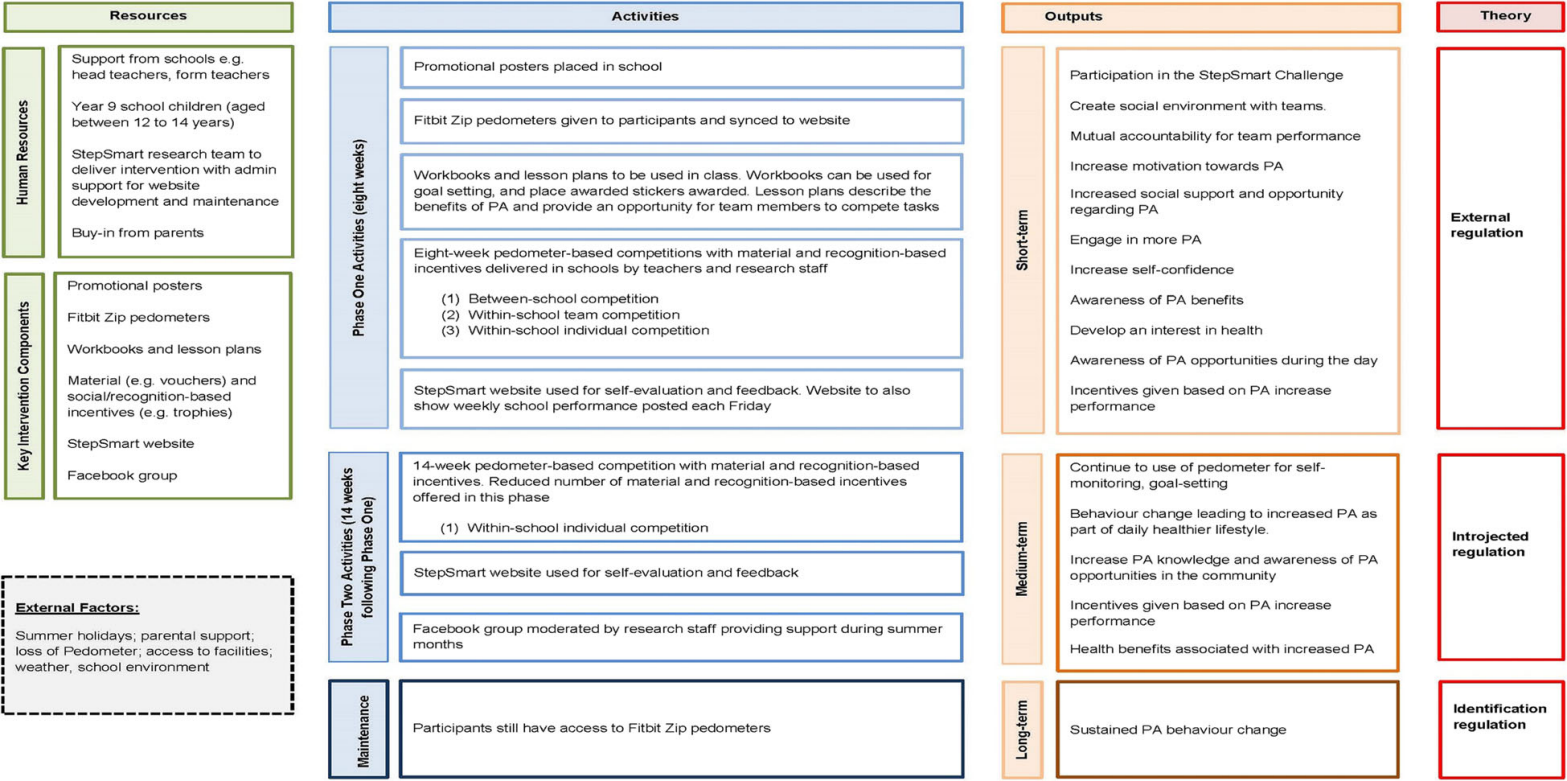
The StepSmart Challenge logic model

The StepSmart Challenge used gamification strategies, combined with the core tenets of self-determination theory, to encourage and support physical activity behaviour change by moving participants along the motivation continuum (from more external forms of motivation, e.g. external regulation) towards intrinsic motivation (e.g. identified regulation) [46], as shown in the intervention logic model (Fig. 1). Researchers visited each intervention school at baseline to collect outcome measures. It was during this time that researchers reiterated (provided initially during the information session) the specific details about what the intervention involved, e.g. explanations were provided about the competition elements, the material and social incentives and The StepSmart Challenge website.

The StepSmart Challenge consisted of two distinct phases (Table 1 shows key intervention components and potential mechanisms). In summary, phase 1 (8 weeks) comprised of competitions on three levels: inter-school, between the three intervention schools; within-school, team-based; and within-school, individual. This phase used a range of material and social incentives to promote physical activity. For the inter-school competition, Fitbit Zip data collected from the three intervention schools was collated to produce an aggregate score for each school. Teachers informed participants on the progress of their school on a weekly basis. This information was also available to participants via The StepSmart Challenge website. At the end of phase 1, the intervention school with the highest number of aggregate steps was awarded a £1000 prize. The within-school team-based competition ran alongside the inter-school competition and involved approximately 10 teams within each school (four to five adolescents within each team). The selection of teams took current physical activity levels and friendship networks into account (obtained from baseline outcome assessments), to ensure that each team included participants with a range of physical activity levels and at least one friend. The aggregate step count of each team was updated weekly on The StepSmart Challenge website leader board. The team with the highest number of aggregate steps within each school at the end of phase 1 were the winners. The team competition was comprised of social incentives such as the publication of results on The StepSmart Challenge website and a trophy which was awarded to the leading team in each school at the end of the competition. The within-school individual competition ran alongside the inter-school, and within-school team-based competitions. Each week, participants within each school competed to be the ‘walker of the week’ (the participant that had accumulated the most steps in the week) or the ‘most improved’ participant (the participant that had increased their step count the most from the previous week). The two weekly winners in each school would receive material incentives such as selfie sticks and vouchers (approximate value of £10) and a social incentive (a certificate of achievement).

**Table 1:** The StepSmart Challenge key intervention components and potential mechanisms

Component	Activity/task	Potential mechanisms
Competition	The participating classes from the three intervention schools joined a pedometer competition using Fitbit Zip pedometers. **Phase 1 (8 weeks)** *Inter-school pedometer competition* Fitbit Zip pedometer data collected across all three intervention schools was collated to produce an aggregate school score. Teachers informed the participants on the progress of their school on a weekly basis. This information was also available to participants via The StepSmart Challenge website. *Within schools team competition* The team competition ran alongside the main school competition and involved approximately 10 teams within each school (five adolescents within each team). The highest placed team within each school at the end of phase 1 were the winners. *Within schools individual competition* Each week, all participants within each school competed to be the ‘walker of the week’ (the participant that had accumulated the most steps in the week) or the ‘most improved’ participant (the participant that had increased their step count the most from the previous week). **Phase 2** *Within schools individual competition* Phase 2 began immediately after phase 1 ended. The inter-school and team competitions were replaced with an individual level competition within each school using the Fitbit Zip pedometers. The individual pedometer competition awarded the three participants in each school who had accumulated the most steps during this phase. Participants were encouraged to complete weekly challenges to increase their steps via The StepSmart Challenge website in this phase (e.g. ‘your weekly mission (should you choose to accept it) will be to walk or run the length of your street at least once every day’). Other missions encouraged participants to go walking with friends and family. This encouraged pro-social behaviour among participants in relation to physical activity outside of a school and team-based competition format.	The use of competition and challenges has been suggested as a way of making a physical activity intervention more engaging and enjoyable, which in turn can help maintain continued participation ([13] [64];).
Team working and social networks (e.g. working in teams for the intra-school competition and inter-school competition)	Selection of teams took current physical activity levels and friendship networks (using the social network data collected at baseline) into account, to ensure that each team (4–5 participants) included participants with a range of physical activity levels and at least one nominated friend.	The effect of peers on influencing physical activity in adolescents has been established ([17] [51];). Behavioural economics suggests this can be harnessed to counteract low levels of self-control [60]. Teams can also provide an opportunity for peer recognition which may increase feelings of self-competence, enjoyment and likelihood of maintained participation ([6] [34];).
Workbooks	A short workbook was given to participants at the start of the intervention. This included ‘fun-facts’, tips and challenges to promote physical activity behaviour individually and as part of a team. There was also as a section for the participant to record weekly step target (individual and team).	Self-determination theory proposes that a sense of relatedness with (the belonging to a group) is a fundamental psychological need for motivation [46]. This can also further foster a sense of connectedness to the team and thus team members could help encourage each other to increase physical activity levels.
Behavioural incentives	**Phase 1 (8 weeks)** *Inter-school pedometer competition* £1000 prize was awarded to the school with the highest aggregated number of steps at the end of the phase. *Within schools team competition* The team competition was comprised of social incentives such as the publication of results on The StepSmart Challenge website, and a trophy which was awarded to the leading team in each school at the end of the competition. *Within schools individual competition* The weekly ‘walker of the week’ and ‘most improved’ received a certificate, and a prize which varied over the course of the competition (e.g. selfie sticks, cinema tickets, gift certificates). **Phase 2 (14 weeks)** *Within schools individual competition* Phase 2 represented a tapered withdrawal from the extrinsically motivated behaviour change techniques towards more intrinsically motivated behaviours. Instead of weekly prizes, the three highest performing participants in each school were each presented with a trophy and a ‘goody bag’ comprising of an assortment of material incentives, e.g. selfie sticks, £10 vouchers. Other incentives were more abstract and took the form of ‘virtual badges’ to represent their achievements; this could be viewed on a participant’s personal profile on The StepSmart Challenge website.	Behavioural incentives contingent on successful performance of a behaviour provide positive reinforcement that can increase the frequency of the behaviour [54]. Behavioural incentives may also work to initiate physical activity in participants with low motivation due to present orientation and high levels of impulsivity [44].
Fitbit Zip pedometers	Participants were given a Fitbit Zip pedometer and asked to wear it every day of the intervention (phase 1 and phase 2). Fitbit Zips provided participants feedback on daily steps, and step data were uploaded to the study website via the Fitbit mobile application or a wireless dongle located at designated areas within schools.	Previous research using pedometers have shown success in increasing children and adolescents physical activity [32]. Pedometers provide real-time feedback. This continual feedback allows individuals to self-regulate behaviour by self-monitoring physical activity [45]. Regular feedback can provide positive feedback and instil feelings of competence when meaningful achievements are reached e.g. self-directed goals [46]. Regular feedback and opportunities to self-monitor behaviour can also counteract low motivation by keeping the activity salient [34].
The StepSmart Challenge website	Fitbit Zip data were uploaded to The StepSmart Challenge website and participants could review their daily/weekly scores and view the competition leader board. The website included the provision of motivational messages, weekly challenges and links to other physical activity resources.	The website provides regular feedback that shows participants their own physical activity in relation to the physical activity achieved by peers. This feedback might help keep the activity salient and motivate participants to increase their physical activity to normative levels (relative to the group) [64].
Facebook group	The Facebook group was created to provide support during the summer months (phase 2). This was a closed group, which was accessible to only The StepSmart Challenge participants from all three intervention schools. As a further protective measure teaching staff could view and moderate all communication via Facebook. This group provided participants with a convenient way to contact research staff, an opportunity to share their progress, and a platform for research staff to suggest different types of physical activity and provide motivational messages.	Facebook is a popular social network site among adolescents. Utilising this platform provides an opportunity for researchers to support and engage with participants, and participants to engage with each other during the summer months. This group was also used so researchers could post different opportunities to increase physical activity in the local area, and for participants to share their physical activity achievements.

Phase 2 (14 weeks) began immediately after phase 1 ended and consisted of a within-school individual pedometer competition. Participants were encouraged to complete weekly challenges to increase their steps via The StepSmart Challenge website. Other missions encouraged participants to go walking with friends and family. This encouraged pro-social behaviour among participants in relation to physical activity outside of a school and the team-based competition format. Phase 2 represented a tapered withdrawal from the extrinsically motivated behaviour change techniques towards more intrinsically motivated behaviours. Instead of weekly prizes, the three highest performing participants in each school were each presented with a trophy and a ‘goody bag’ (approximate value of £30, consisting of an assortment of material incentives used in phase 1). Other incentives were more abstract and took the form of ‘virtual badges’ to represent their achievements; this could be viewed on a participant’s personal profile on The StepSmart Challenge website. Before the start of phase 2, participants were invited to join a closed Facebook group. This provided participants with a convenient way to contact research staff an opportunity to share their progress and a platform for research staff to suggest different types of physical activity and provide motivational messages.

### Control

Control participants did not receive any form of intervention. The participants were not provided with Fitbit Zip pedometers, and they did not take part in the competitions and did not receive material or social incentives during the intervention period. Control participants were asked to complete the same outcome assessments as the intervention group at each time point. Control group schools were each given £400 to cover the cost of staff time associated with participation in the study.

### Outcome measures

#### Recruitment and retention

The numbers of schools and adolescents who were invited to participate and consented were recorded at T0 (baseline). The numbers of schools and participants who withdrew, were lost to follow up and retained were recorded post-intervention, at 22 weeks (T1) and T2 (52 weeks).

#### Proposed outcome measures

Minutes of daily moderate-to-vigorous physical activity (MVPA) were measured using validated ActiGraph GT3X/GT3X+ accelerometers (ActiGraph Inc., USA). Accelerometers were provided to participants at T0, T1 and T2 to wear for 7 consecutive days and to only remove it when bathing, swimming or sleeping. Activity counts using the accelerometers were recorded in 1 s epochs. In order to obtain total minutes of MVPA per day, the data were reintegrated in 60 s epochs before Evenson cut off points were applied to the data [16]. Non-wear time was defined as a run of zero counts lasting more than 60 min [5]. Valid data were defined as (a) a minimum of 8 h/day wear-time and (b) for at least 3 days [2, 27]. At each time point, participants at each school completed paper-based questionnaires in their respective classrooms. The questionnaires included the Warwick-Edinburgh Mental Wellbeing Scale (WEMWBS) [59], perceived social support for physical activity [49], social networks [19] and a future orientation scale to measure time preferences [42]. Class teachers completed the Strength and Difficulties Questionnaire (SDQ) at their desk for each participant in their classroom, while participants were completing the paper-based questionnaires that they were provided. The SDQ is used to assess emotional and behavioural problems and pro-social behaviour [18].

Outcome measures were administered by members of the research team (RC, PB, RON, MT and RH) during agreed scheduled classes on the school premises. Teachers completed a Strength and Difficulties Questionnaire for each participating pupil in their class while the pupils were completing their questionnaires. To explore the acceptability of the intervention and study design, focus groups were conducted at all the above time points and at the end of phase 1(8 weeks post-baseline) (Table 2).

**Table 2:** Details of the outcome measures

Tool	Outcome	Derived variable(s)	Mode of completion	Time point
ActiGraph GT3X/GT3X+ monitors	Minutes of daily MVPA	Minutes of daily MVPA (physical activity ≥ 2296 counts per minute)	Accelerometer provided to participant to wear for 7 consecutive days and to only remove it when bathing, swimming or sleeping.	T0, T1, T2
	Minutes of daily light physical activity	Minutes of daily light physical activity (101 to 2295 counts per minute)		
			Activity counts using the accelerometer were recorded in one second epochs. In order to obtain total minutes of MVPA per day, the data were reintegrated in 60 s epochs before Evenson cut off points were applied to the data [16]. Non-wear time was defined as a run of zero counts lasting more than 60 min [5].	
	Minutes of daily moderate intensity physical activity	Minutes of daily moderate intensity physical activity (2296–4011 counts per minute)		
		Minutes of daily sedentary time (≤ 100 counts per minute)		
	Minutes of daily sedentary time			
			Valid data were defined as: (a) a minimum of 8 h/day wear-time; (b) for at least three days ([2] [27];).	
Warwick-Edinburgh Mental Wellbeing Scale [55]	Mental wellbeing	Level of mental wellbeing (sum of 14 items)	Paper-based questionnaire completed by participants.	T0, T1, T2
Social support questions from previous research ([40] [49];)	Perceived social support for physical activity	Parental support (average of items 6A, 6B and 6C)	Paper-based questionnaire completed by participants.	T0, T1, T2
		Parental encouragement (average of items 6D and 6E)		
		Teacher support (average of items 6F, 6G and 6H)		
		Peer support (average of items 6I, 6J,and 6K)		
Social networks [19]	Friendship networks	Friendship networks (who in your class are you friendly with or hang about with? You can list up to five names)	Paper-based questionnaire completed by participants.	T0, T1, T2
		Team captain (who do you respect/look up to in your class? Provide first name and surname of one classmate)		
Future orientation scale [42]	Time preferences	Planning horizon score (sum of items 1, 4, 5 and 7)	Paper-based questionnaire completed by participants.	T0, T1, T2
		Impulsivity score (sum of items 2, 3, 6 and 8)		
Strength and Difficulties Questionnaire [18]	Emotional and behavioural problems	Pro-social (sum of items 1, 4, 9, 17 and 20)	The research team provided class teachers a paper-based Strength and Difficulties Questionnaire (SDQ) to complete at the time participant data collection. The research team collected the SDQs from teachers If they completed the SDQs during this period. If teachers required more time, a date was arranged for the a member of the research team to return the school and collect questionnaire	T0, T1, T2
		Peer problems (sum of items 6, 11, 14, 19 and 23)		
		Hyperactivity/inattention (sum of items 2, 10, 15, 21 and 25)		
		Conduct problems (sum of items 5, 7, 12, 18 and 21)		
		Emotional symptoms (sum of items 3, 8, 13, 16 and 24)		
		Total difficulties score (sum of all scales except pro-social scale)		

### Intervention acceptability

Repeated semi-structured focus groups were conducted with a subsample of adolescent participants in each school. The same participants were invited at each focus group. Participants were recruited using purposive sampling in consultation with teaching staff. Teachers identified potential participants with a range of physical activity levels (low to high) as well as mixed educational ability. Three focus groups were conducted in each intervention school (at T0, T1 and T2), and two focus groups were conducted in each control school (at T0 and T2). Participants (*n* = 33) were recruited using purposive sampling in consultation with teaching staff. Teachers identified potential participants with a range of physical activity levels (low to high) as well as mixed educational ability. The same participants were invited at each focus group; however, the number of participants varied due to school absences (mean of 6 participants; range 2–7 participants). No participants refused to take part or dropped out of the focus group discussions. Focus groups were conducted by PB, RC and RO’N. RC was present at all focus groups, and either PB or RO’N supported RC by taking field notes at each session. The focus groups lasted on average 33 min (range 21–41 min) and were audio-recorded and transcribed verbatim. The topic guide was refined iteratively following interim analysis at each data collection point. Additional file 1 details the topic guide for the focus groups.

At T2, a focus group was also conducted with three teachers from intervention schools. This focus group was audio-recorded and transcribed verbatim.

### Analyses

Proportions were used to describe the recruitment rates (for schools and participants) and the retention rates at T1 and T2. Descriptive analyses were performed using the Statistical Package for Social Sciences (SPSS) version 22.0 (SPSS Inc., Chicago, USA). Due to the non-normal distribution of the accelerometer data, median and interquartile ranges (IQR) were calculated for these outcomes. Accelerometer data were processed using ActiLife version 6.13.1 (ActiGraph Inc., USA). Imputation analysis using last observation carried forward (LOCF) was conducted to treat missing data [21] for the accelerometer data. Outcome data from WEMWBS and SDQ were normally distributed and were therefore presented as mean and standard deviation (SD). All other outcomes were non-normally distributed and presented as median and IQR. Exploratory analysis was conducted on the association between friendship within teams and physical activity, using R version 3.3.2. Accelerometer data were processed using the ActiGraph software package version 1.0.1 [11]. Feasibility studies are not designed to investigate the effectiveness of interventions [29]. Thus, no significance testing was conducted.

### Intervention acceptability

All focus group transcripts were imported into NVivo (Version 10, QSR, Southport, UK) to manage and support analysis using the thematic analysis framework [4]. Initially, researchers (RC and PB) familiarised themselves with the data. RC and PB developed a sample coding frame independently and this was refined iteratively with subsequent discussions. Anonymised illustrative quotes supporting emerging themes were highlighted and agreed by researchers.

## Results

### Aims 1 and 2: to test the feasibility of recruiting and retaining schools and adolescents in The StepSmart Challenge

**Fig. 2:**
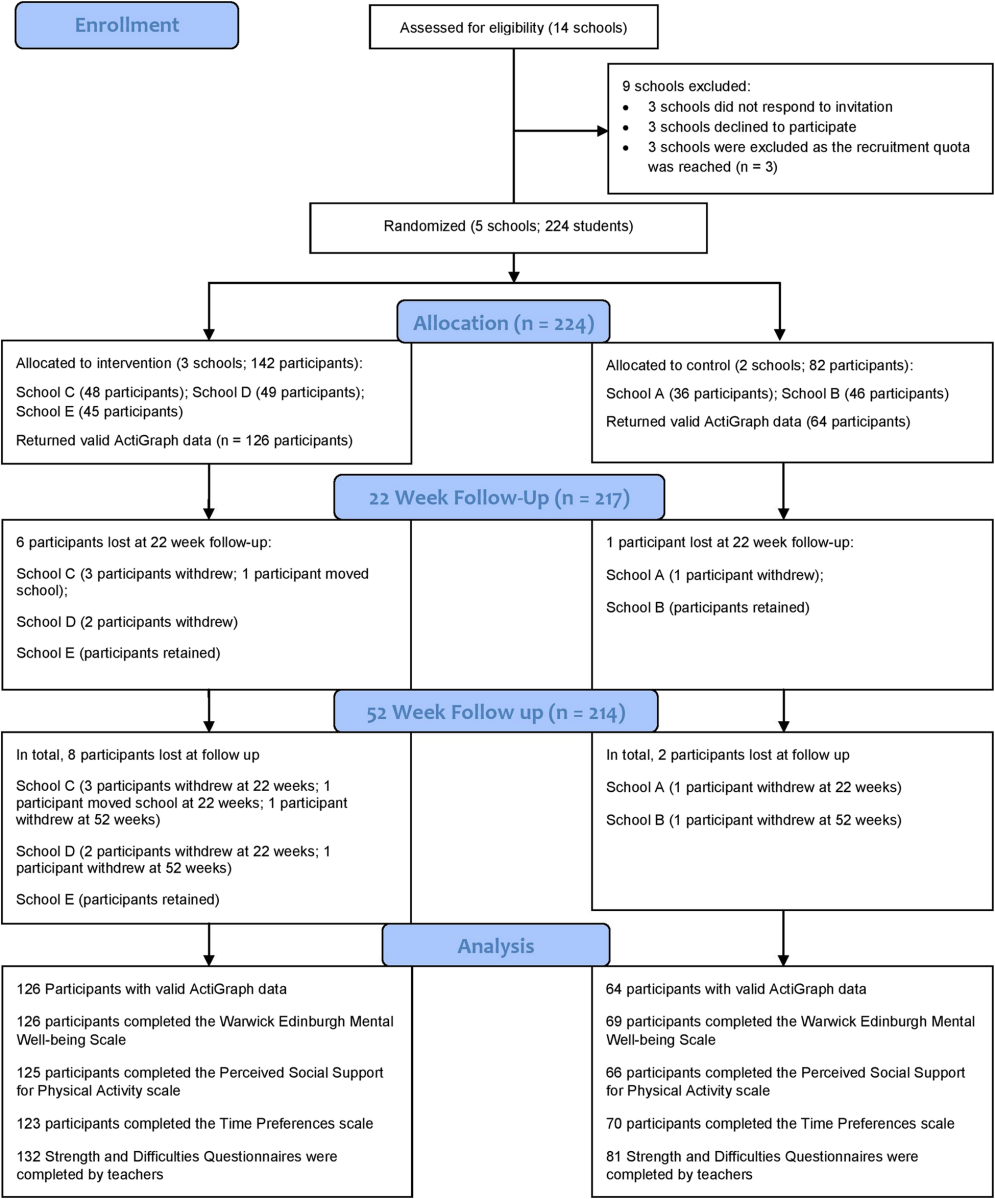
CONSORT participant flow diagram

Fourteen schools were approached to participate in the study (see Fig. 2). Schools were given 1 week to respond before a follow-up phone call was made by a member of the research team. In total, 11/14 schools responded: 8 were interested in participating and we recruited the first 5 of these respondents. Three other respondents declined to take part for the following reasons: (1) already committed to other research projects, (2) staff pressures and (3) timetabling issues. Baseline characteristics of participating schools are shown in Table 3. Most participating schools were single-sex schools. Three schools had high levels of free school meal eligibility (> 37.5%). Of the 236 students who were invited to participate, 224 (94.9%) provided written consent.

**Table 3:** Characteristics of participating schools

	Intervention or control	Free school meal eligibility (%)	Single-sex or co-educational school	Total Year 9 pupils in school (*n*)	Potential participants invited (*n*)	Participants at baseline (*n*)	Participants retained at 52 weeks (*n*)
School A	Control	Lower SES (63.7)	Single sex (male)	43	40	36	35
School B		Higher SES (7.2)	Co-educational	254	48	46 (25 females)	45
School C	Intervention	Higher SES (8.0)	Single sex (male)	160	48	48	44
School D		Lower SES (56.5)	Single sex (female)	68	50	49	46
School E		Lower SES (54.6)	Single sex (female)	151	50	45	45
Total					236	224 (53.1% female)	215

### Aim 3: to assess feasibility of using the proposed outcome measures

At T0, 214/224 (95.5%) participants returned accelerometers, of which 190/224 (84.8%) of participants returned valid data for minutes/day of MVPA (Additional file 2 shows data by school). At T2, 198/214 (92.5%) participants returned accelerometers; 123/214 (57.4%) returned valid data for analysis. Overall, almost half, 21/44 (47%), of the unreturned devices were attributed to school A. School A was assigned to the control group and was based in an area of high disadvantage. Table 4 shows the completion rates for all proposed outcomes. The completion of WEMWBS ranged from 213/224 (95.0%) at T0 to 195/224 (87.0%) at T2. Response rates for SDQs from teachers were low (65%) during T0 due to staff absence within one school.

**Table 4:** Data collected for proposed outcome data

Outcome	T0 (Baseline)		T1 (22 weeks)		T2 (52 weeks)	
	Intervention	Control	Intervention	Control	Intervention	Control
Accelerometer outcomes	*n* = 126, median (IQR)	*n* = 64, median (IQR)	*n* = 126, median (IQR)	*n* = 64, median (IQR)	*n* = 126, median (IQR)	*n* = 64, median (IQR)
MVPA (min/day)	33.3 (23.6–49.0)	43.6 (31.0–69.3)	33.0 (20.0–46.2)	47.4 (32.7–65.1)	33.4 (19.2–49.2)	37.2 (26.5–53.1)
Light intensity PA (min/day)	336.1 (232.4–435.6)	306.2 (237.0–422.6)	225.4 (175.2–283.2)	236.1 (183.2–295.1)	201.0 (167.6–256.0)	189.5 (145.6–241.8)
Moderate intensity PA (min/day)	27.1 (18.2–37.7)	35.1 (25.2–49.8)	25.8 (17.1–33.2)	33.4 (25.4–44.2)	25.0 (16.2–34.8)	29.1 (21.7–38.6)
Vigorous intensity PA (min/day)	6.6 (3.0–11.9)	8.6 (3.3–18.7)	6.2 (2.4–11.8)	12.2 (5.6–19.8)	5.9 (2.0–13.0)	7.4 (4.5–13.8)
Daily sedentary time (min/day)	469.6 (416.5–524.3)	466.3 (410.0–534.9)	454.7 (405.7–517.8)	453.5 (399.8–529.6)	461.9 (400.6–519.4)	462.9 (399.2–531.3)
Steps per day	8019 (6549.6–9738.9)	9160 (7485.5–11,975.5)	8298 (6370.5–10,641.0)	11,162 (7957.7–13,888.4)	7064 (5620.3–9440.0)	7856 (5847.1–12,473.8)
Wellbeing	*n* = 139, mean (SD)	*n* = 74, mean (SD)	*n* = 124, mean (SD)	*n* = 71, mean (SD)	*n* = 126, mean (SD)	*n* = 69, mean (SD)
WEMWBS	51.8 (8.4)	51.9 (8.3)	50.0 (9.1)	50.1 (10.9)	51.1 (9.0)	49.3 (10.4)
Strength and difficulties	*n* = 134, mean (SD)	*n* = 22, mean (SD)	*n* = 133, mean (SD)	*n* = 35, mean (SD)	*n* = 132, mean (SD)	*n* = 81, mean (SD)
SDQ	4.7 (5.1)	13.6 (8.1)	4.1 (5.2)	12.5 (6.9)	6.5 (5.1)	7.5 (9.0)
Pro social sub scale	8.5 (1.5)	6.4 (2.8)	8.8 (1.8)	4.7 (2.9)	8.4 (2.0)	7.0 (2.7)
Perceived social support	*n* = 137, median (IQR)	*n* = 75, median (IQR)	*n* = 130, median (IQR)	*n* = 67, median (IQR)	*n* = 125, median (IQR)	*n* = 66, median (IQR)
Peer support	3.3 (3.0–3.6)	3.0 (2.6–3.6)	3.0 (2.6–3.3)	3.0 (2.6–3.3)	3.0 (2.6–3.3)	3.0 (2.3–3.3)
Parental support	2.6 (2.3–3.0)	2.8 (2.3–3.0)	2.6 (2.3–3.0)	2.6 (2.3–3.0)	2.6 (2.3–3.0)	2.6 (2.3–3.0)
Parental encouragement	3.5 (3.0–4.0)	3.5 (3.0–4.0)	3.5 (3.0–4.0)	3.5 (3.0–4.0)	3.5 (3.0–4.0)	3.0 (2.5–4.0)
Teacher support	2.3 (2.0–2.6)	2.3 (1.7–2.6)	2.3 (2.0–3.0)	2.3 (1.6–3.0)	2.3 (1.6–2.6)	2.0 (1.6–2.6)
Time preferences	*n* = 122, median (IQR)	*n* = 62, median (IQR)	*n* = 119, median (IQR)	*n* = 71, median (IQR)	*n* = 123, median (IQR)	*n* = 70, median (IQR)
Planning horizon	7.0 (6.0–8.0)	7.0 (5.0–8.0)	6.0 (6.0–8.0)	6.0 (5.0–8.0)	7.0 (5.0–8.0)	7.0 (5.7–8.0)
Impulsivity	6.0 (5.0–8.0)	6.0 (5.0–8.0)	6.0 (5.0–7.0)	5.0 (4.0–7.0)	6.0 (5.0–7.0)	6.0 (4.0–7.0)

All other outcomes were non-normally distributed and presented as median and IQR. Exploratory analysis was conducted on the association between friendship within teams and physical activity, using R version 3.3.2 (The R Foundation for Statistical Computing., 2016). The accelerometer data was processed using the ActiGraph software package version 1.0.1 [11]. Feasibility studies are not designed to investigate the effectiveness of interventions [29]. Thus, no significance testing was conducted

Table 4 shows the potential for change using the proposed outcomes (for the social network analysis, see Additional file 2). Daily minutes of MVPA (our proposed primary outcome) for the intervention group remained unchanged from baseline at T1 and T2. In the control group, there was a slight increase from baseline in MVPA at T1 (47.4 min/day; IQR 32.7 to 65.1), which decreased closer to baseline levels at T2 (37.2 min/day; IQR 26.5 to 53.1). The data from all other outcomes remained relatively stable at each point.

At each time point, between two and six researchers facilitated data collection in each school depending on staff availability, with PB, RC or R’ON present on every occasion to lead the collection process. Data collection in each school would occur at a prearranged time and day with the majority of participant questionnaire data collected at this time. On occasion, it would be necessary to return to schools to collect questionnaire data for participants who were absent during the data collection session. During the data collection sessions, teachers were provided with the SDQ’s to complete. However, it was not always possible for teachers to complete these during the time researchers were present in the school. In such cases, a researcher would return to the school to collect the SDQ’s a short time after the data collection visit. At each data collection period, researchers were often required to visit each school multiple times to collect accelerometers due to participant absences, participants forgetting to bring them into school or having misplaced the accelerometers. At each time point, accelerometer data was downloaded and processed by one researcher. Accelerometer data was downloaded and processed at each time point in batches (by school), and it would take approximately an half a day to complete the process for each school. At each time point, two researchers were involved in pedometer data collection and analysis.

### Aim 4: to explore intervention acceptability and identify any refinements that could be made to The StepSmart Challenge

Our focus group findings are reported under three main themes: participant acceptability of the intervention, teachers’ perception of relevance of intervention and key implementation findings in The StepSmart Challenge.

#### Participant acceptability of key intervention components

Generally, participants spoke positively regarding taking part in The StepSmart Challenge. In particular, the competition format and incentives on the offer appeared to encourage participation, ‘Every week cos you know it’s like running out of time for like the prizes, [and I] just really want to get one’ (school C, male, T1). However, for some, the competitive nature of the intervention appeared to be viewed as ‘off putting’ as one participant noted, ‘It’s just sort of cause you knew you probably were not going to win so you are just like there’s really no point in wearing [the FitBit Zip pedometer]’ (school E, female, T2). This was despite the presence of a weekly prize for the ‘most improved’ participant.

Team-based competition appeared to be an acceptable approach to encouraging physical activity among participants with qualitative data suggesting a significant role played by peer networks, both in terms of pro-social behaviour ‘when you have your friend with you…you’d be more encouraged to do more walking’ (female, school D) and goal setting ‘you’re just trying to beat your friends’ (male, school C, T1). However, team-based competition was not without its caveats. While some participants highlighted its benefits, ‘if they [team mates] want to go for a run you will want to go for a run with them’ (school C, male, T0), there were some issues in relation to the compositions of teams. Girls, in particular, showed a preference for team(s) based on current friendship networks rather than on a mix of ability in relation to PA; ‘It would have been easier if we were with our friends’ (school D, female, T1) and if ‘you don’t like people in your team you’re just going to be like…[I’m] not even going to talk to you’ (school E, female, T2).

Participants’ comments indicated that they did not use The StepSmart Challenge website regularly and many preferred using the Fitbit mobile application or the display on the Fitbit Zip to track scores; ‘I just didn’t think there was any point cause like you could see it on the pedometer’ (school D, female, T2) and ‘I think loads of people used the app [Fitbit] much more. Some people probably didn’t even go onto the website’ (school D, female, T2). Pupils appeared to engage less with the workbook, preferring to use the Fitbit application instead.

#### Teachers’ perceptions of relevance of intervention

The gender component of the competition was reflected upon within teacher interviews; ‘Boys probably wanted it more for the competition element…their friendship groups at our place [school] are pretty much made up with who they play sport with. Sport’s a massive social thing in our place [school] as well. So, they want to beat their mate, not be in a group with them’ (school C, teacher 1). While teachers representing the girls’ schools stated that they were grateful to be involved in this type of study, they acknowledged the number of girls who regularly engage in sport was relatively minor: ‘What we were finding at our sports days was the usual girls were winning everything’ (school D, teacher 1).

Teachers suggested that the incentives were important to encourage engagement but felt that the value of prizes could be reduced in favour of being able to offer them more often; ‘Scaling down maybe the prizes to make more prizes but at a lesser value… this is not to be nasty to children, but they’ll be impressed by nearly anything.’ (school C, teacher 1). Moreover, providing incentives for reaching a certain level was also suggested, ‘[If] they have reached that level…then you’re rewarding more children than just the one, [they] deserve a reward’ (school C, teacher 2). Teachers suggested that while the workbook was useful, it felt too much like homework to pupils, ‘I think they were easy enough to use …[but] they see that as work; they don’t see that as that’s just a sheet or a book, no, it’s work’ (school C, teacher 1).

The StepSmart Challenge also appeared to go beyond physical health and physical activity and was thought of more as a ‘wellbeing’ or ‘social’ intervention by teachers. As one teacher noted, ‘Some of it could be maths, art or whatever, but it’s that sort of feeling of group togetherness…whether it’s well-being mentally or well-being physically, [can have] an impact on them’ (school C, teacher 1). This view was supported within another school: ‘I think yours [StepSmart] has provided a starting point for us…that we can take it forward and look at the whole physical and mental health of pupils’ (school E, teacher 2).

#### Future implementation

Several participants suggested that wrist-worn devices for monitoring physical activity could reduce loss and increase wear time; ‘I think…you can get those [pedometers] in your wrists but they are more expensive and maybe a lot better like but for people our age… it’s like a lot like getting changed after the shower etc. so we always forgot to like put it on.’ (school C, male, T2). This preference for wrist-worn devices may also extend to the wearing of accelerometers for measuring physical activity; ‘Something else maybe that you can just put on your wrist might be a little bit easier’ (school D, female, T1) and ‘It’s the fact that you could see it like with a crop top [when] you’re walking’ (school E, female, T2).

Further, it was suggested that The StepSmart Challenge may be expanded to other subjects out with Physical Education (P.E.), ‘It worked very well in the P.E. department, but I can see how it can lend itself to other departments in relation to whether it’s the maths department, the science department, which would be good’ (school E, teacher 1).

Regarding implementation, it was noted that parental opt-out consent was a particularly valuable and welcomed approach, ‘Opt out I find, when you’re dealing with children and parents, is always better’ (school A, teacher 2) and ‘It’s [parental opt-out] easier because they don’t have to sign anything’ (school B, teacher 1). Teachers felt this significantly reduced their administration burden and the task of ‘chasing up’ outstanding parental consent forms.

## Discussion

Our findings suggest that The StepSmart Challenge, a gamified physical activity intervention, is acceptable to adolescents aged 12–14 years. Recruitment and retention rates of schools and participants indicate that it is feasible to scale up the intervention for an effectiveness trial. In general, the questionnaires were well understood and completed at each time point and were deemed appropriate for use in a future study. However, The StepSmart Challenge would benefit from the refinement of the website used and participant workbooks, and there is a need to explore how to maximise the return of valid accelerometer data from participants, as well as questionnaire data from teachers.

Participant recruitment and retention was high in both the intervention and control groups. A contributing factor to participant recruitment was the use of parental opt-out consent. Tigges [61] noted an increase of approximately one third when choosing passive (opt-out) as opposed to active (opt-in) parental consent. Extensive discussions took place with the schools regarding the process of parental opt-out consent, but we recognise that this may depend on the nature of the intervention. It has also been suggested that passive consent is more inclusive of those less engaged within the school system or those from ethnic minority backgrounds [61]. A limitation of opt-out consent was not having the contact details of parents or participants in order to send reminders or prompt wearing of accelerometers. This disadvantage in using opt-out consent has also been shown in previous research by [8].

Approaches to improve the return of accelerometer data require further consideration before scaling up for a full trial. Difficulties were encountered within one of the control boys schools which accounted for 11/35 (31.4%) of accelerometers not returned and of those who returned only 9/24 (37.5%) provided valid data. A range of additional efforts was made to try to improve retention rates and the return of devices, such as additional visits to the schools by the research team, letters sent to parents from the school principal and offering vouchers for the return of devices. Participants in the control schools did not receive any intervention nor were they on a waiting list to receive the intervention. Therefore, participants may not have felt particularly incentivised to complete the outcome measures. Future research could explore the use of wrist-worn accelerometers or increasing the value of vouchers provided on return of accelerometers. Many post-primary schools have adopted a text messaging service to relay information to parents. Utilising this system to deliver prompts could be one way to improve participant wear time and the return of accelerometers but would inevitably impose further burden on the schools.

Research by Hunter et al. [23] suggests that competition may extrinsically motivate and encourage physical activity behaviour among school children. The findings from our qualitative data reported in a previous publication [9] highlighted that participants felt competition could provide a sense of enjoyment, feelings of competence and wellbeing which is consistent with the self-determination theory. Building on this, teacher focus groups proposed the benefit of changing the incentive structure in the competition which would allow more participants to be rewarded. This positive feedback might increase engagement in participants and encourage long-term physical activity behaviour change. Implementing The StepSmart Challenge as part of a health promotion programme embedded in the school curriculum could be a method to add the physical activity education and knowledge acquisition elements of The StepSmart Challenge without increasing the burden on teachers [41]. This holistic approach to The StepSmart Challenge was supported by teachers who felt that a lifelong learning approach to The StepSmart Challenge would be needed and to get the buy-in from senior staff in school [43].

A novel aspect of this school-based study was the purposeful effort to continue the study across the summer months. This was made possible by The StepSmart Challenge website and Fitbit application, through which participants could track and monitor their progress, as well as complete challenges and receive virtual reward badges (phase 2). Continuation over the summer holidays was viewed as important to try and maintain healthy physical activity habits outside of the school context. The accessibility of the application (via mobile phones) appeared to be preferable to using the study website and qualitative data suggested that most participants who were actively engaged in the study uploaded pedometer data via the Fitbit application.

## Conclusions

As young people’s lives become more intertwined with technology, the use of innovative gamified interventions could be one approach to engage and motivate behavioural change in this population. Results from this feasibility study have provided support for the acceptability of an intervention that incorporates such approaches. The study also demonstrated the suitability of the proposed school and participant recruitment methods and using parental opt-out procedures. Further thought must be given to how we boost accelerometer retention in future studies and how the intervention can be embedded within the school curriculum. Incorporating process evaluations and qualitative research in future research would add further depth to our understanding of outcome evaluations and provide insight into the intervention’s external validity which is necessary before decisions are made to scale up public health interventions.

## Supplementary information

**Additional file 1:** **Table S1.** Focus groups topic guide.

**Additional file 2:** **Table S2.** Accelerometer data for participating schools. **Table S3.** Proportion of nominated friends in teams. **Figure S1.** MVPA (minutes) at baseline and 6 months: relationship with friendship density in team. **Figure S2.** Change in minutes of MVPA from baseline to 6 months: relationship with friendship density in team.

## Data Availability

The datasets generated and/or analysed during the current study are not publicly available due to the requirement from the ethics review committee but are available from the corresponding author on reasonable request.

## Abbreviations

BCTs: Behaviour change techniques
CPM: Counts per minute
IQR: Interquartile range
LOCF: Last observation carried forward
MVPA: Moderate to vigorous physical activity
NICE: National Institute for Health and Care Excellence
PE: Physical Education
SD: Standard deviation
SDQ: Strength and Difficulties Questionnaire
SDT: Self-determination theory
SPSS: Statistical Package for Social Sciences
T0: Baseline
T1: End of phase 2 (22 weeks post-baseline)
T2: Follow up (52 weeks post-baseline)
WEMWBS: Warwick-Edinburgh Mental Wellbeing Scale
