# Supplementary material for: A feasibility study of ‘The StepSmart Challenge’ to promote physical activity in adolescents
Source: Pilot Feasibility Stud. 2019 Nov 17;5:132. doi: 10.1186/s40814-019-0523-5 (PMC6859606; doi:10.1186/s40814-019-0523-5)
Supplement: Supplementary file 1 — Additional file 1: Table S1. Focus groups topic guide. [file 40814_2019_523_MOESM1_ESM.docx]

# Supplementary File 1

**Table S1: Focus groups topic guide**

| **Topic Guide** | | | |
| --- | --- | --- | --- |
| **Pre-intervention (T0)** | **End of phase one (T1)**  **(External regulation; Introjected regulation)** | **End of phase two (T2)**  **(Introjected regulation; Identified regulation)** | **12 months post-baseline follow up (T3)**  **(Integrated motivation)** |
| **Explore the barriers and facilitators to PA**  What stops you from being physically active? (Relational support)  Role of parents/friends in your PA? (Autonomy support; Relational support)  Any ideas to increase PA? (Autonomy support)  Opportunities for PA within school/community? (Autonomy support; Relational support) | **Explore the experiences of The StepSmart Challenge**  How easy is it to find ways in which to be more active? (Autonomy support)  Did completing The StepSmart Challenge with friends make it easier? Or more pressure? (Autonomy support; Relational support)  Do you enjoy telling people about your success in terms of PA (e.g. showing others your steps)? (Perceived competence; Relatedness) | **Explore how the participants felt about the different competition elements**  How did the team competition compare to the individual competition? (Relational support)  Some of the class were more/less active than you. How did you feel when you compared your steps to the class? (Perceived competence)  Did team members encourage you to be active or not? (Autonomy support)  How did you feel if you did not contribute to the team/school’s step count? (Perceived competence) | **Explore if there has been a change in PA (formation of habit)**  One year on: how active are you now (compared to before you took part)?  Did you make changes to your PA? Why/why not? *(changes to routine ... active travel, walking with friends etc.)*  If you did make any changes, which are you still doing? What strategies helped? (Autonomy support) |
| **Explore the acceptability of the intervention components**  Have you ever heard of…or used a pedometer?  What is it you (dis)like about them?  Feelings on writing down your own daily steps goals in a workbook. (Perceived competence; Autonomy support)  What features of a website would you find appealing?  How do you feel about entering a school-based competition?  Any advantages/disadvantages to team vs individual competitions  What prizes would motivate young people to take part? | **Explore the attitude towards PA**  Do you enjoy PA?  What are your main reasons for being active? (i.e. role of the prizes and other elements?)  Do you enjoy finding new ways of becoming more active? (Autonomy support)  Do you think your motivation towards PA has changed? | **Explore how The StepSmart Challenge instigated any changes in participants PA**  How do you feel about PA since The StepSmart Challenge?  What have you enjoyed?  Has The StepSmart Challenge increased your PA? If not, what would motivate you to be more active?  Since The StepSmart Challenge, what new opportunities have you found to be active? (Autonomy support)  Anything outside your control stopping you from being active? (e.g. park proximity, family, other) (Autonomy support; Relational support) | **Explore how participants felt about The StepSmart Challenge**  Why did you choose to participate?  Good/not so good aspects of the competition?  Did the competition motivate – no/at beginning/all throughout? |
|  | **To explore the consequences of PA**  Do you find yourself having more energy?  Do you feel more confident in being able to achieve things? (Perceived competence)  How do you feel after you have completed some PA?  Do you think you are more active now than when you started? Why? | **Explore how participants felt about the use of the pedometer**  How often did you use the pedometer? What did you like/dislike about it? (e.g. Feedback) (Perceived competence; Autonomy support)  How many of you are still using the pedometer? Why? | **Explore how participants felt about the team/individual competition**  Class vs school, was one enjoyed more? (Autonomy support; Relational support)  Influence of friends on being active? (Autonomy support; Relational support)  Opportunity to make new friends? (Relational support)  Feelings about not contributing to the team/school step count? (Perceived competence)  More motivated if friends wanted to do well in the competition? (Relational support) |
|  |  | **Explore how participants felt about the rewards/prizes**  What did you think of the prizes?  Tell me how well they worked to motivate you?  How did you feel if you did not win a prize? (Perceived competence)  If no prizes, but still a competition, would you still be as active? Why? | **Explore how participants felt about the rewards/prizes**  Type of prizes preferred, including trophy or a certificate?  Not competing against others, but beating your own goal? (Autonomy support; Perceived competence) |
|  |  | **Explore how the website and Facebook group were used**  Things you liked/did not like about the website?  What did you think of Facebook group? (Autonomy support; Relational support)  Which way would you prefer to get your information? (Autonomy support) | **Mood/motivation did this change at different stages of the intervention?**  Feelings if did not make as many steps as others? Did that affect your mood? (Perceived competence; Relational support)  If you did/did not do well - did that motivate/demotivate you? (Perceived competence; Relational support)  If you felt like you did not have a good chance to win would that make you stop trying? (Perceived competence; Relational support) |

Red text illustrates how items in the topic guide are linked the concepts of self-determination theory
