# Supplementary material for: A feasibility study of ‘The StepSmart Challenge’ to promote physical activity in adolescents
Source: Pilot Feasibility Stud. 2019 Nov 17;5:132. doi: 10.1186/s40814-019-0523-5 (PMC6859606; doi:10.1186/s40814-019-0523-5)

# Supplementary File 2

**Table S2: Accelerometer data for participating schools**

| **T0 (Baseline)** | **Number of ActiGraphs given** | **Number of ActiGraphs returned** | | **Number of ActiGraphs not returned** | **ActiGraphs with valid data** | **ActiGraphs with non-valid data*** | **Number of** **participants not given ActiGraphs** e.g. due to absence at time of data collection |
| --- | --- | --- | --- | --- | --- | --- | --- |
| School A | 36 | 33 | | 3 | 22 | 10 (M = 10) | 0 |
| School B | 46 | 45 | | 1 | 42 | 3 (M = 2; F = 1) | 0 |
| School C | 48 | 48 | | 0 | 45 | 3 (M = 3) | 0 |
| School D | 49 | 48 | | 1 | 43 | 5 (F = 5) | 0 |
| School E | 45 | 40 | | 5 | 38 | 2 (F = 2) | 0 |
| **Total T0** | **224** | **214 (95.5%)** | | **10 (4.4%)** | **190 (84.8%)** | **23 (10.2%)** | **0** |
|  | | |  | | | | |
| **T1 (22 weeks)** | **ActiGraphs given** | **ActiGraphs returned** | | **ActiGraphs not returned** | **ActiGraphs with valid data** | **ActiGraphs with non-valid data*** | **Lost to follow up at T2** |
| School A | 35 | 28 | | 7 | 16 | 12 (M = 12) | 1 |
| School B | 46 | 42 | | 4 | 36 | 6 (M = 4; F = 2) | 0 |
| School C | 45 | 40 | | 5 | 35 | 5 (M = 5) | 3 |
| School D | 47 | 46 | | 1 | 23 | 13 (F = 13) | 2 |
| School E | 45 | 45 | | 0 | 29 | 16 (F = 16) | 0 |
| **Total T2** | **218** | **201 (92.2%)** | | **17 (7.7%)** | **139 (63.7%)** | **52 (23.8%)** | **6** |
|  | | |  | | | | |
| **T2(52 weeks)** | **ActiGraphs given** | **ActiGraphs returned** | | **ActiGraphs not returned** | **ActiGraphs with valid data** | **ActiGraphs with non-valid data*** | **Lost to follow up at T3** |
| School A | 35 | 24 | | 11 | 9 | 15 (M = 15) | 1 |
| School B | 45 | 45 | | 0 | 27 | 18 (M = 10; F = 8) | 1 |
| School C | 44 | 40 | | 4 | 31 | 9 (M = 9) | 4 |
| School D | 46 | 45 | | 1 | 27 | 18 (F = 18) | 3 |
| School E | 45 | 44 | | 1 | 29 | 15 (F = 15) | 0 |
| **Total T3** | **215** | **198 (92.0%)** | | **17 (7.9%)** | **123 (57.2%)** | **75 (34.8%)** | **9** |
|  | | |  | | | | |
| **Overall, across 3 time points** | **ActiGraphs given** | **ActiGraphs returned** | | **ActiGraphs not returned** | **ActiGraphs with valid data** | **ActiGraphs with non-valid data*** |  |
| **Total N (%)** | **657** | **613 (93.3%)** | | **44 (6.6%)** | **452 (68.7%)** | **148 (22.5%)** |  |

*Non-valid wear time was defined as a run of zero counts lasting more than 60 minutes (Cain and Geremia, 2012). Valid data were defined as: (a) a minimum of 8 hours/day wear-time; (b) for at least three days (Bingham et al., 2016; Jago et al., 2015).

M = Male Participants; F = Female participants

# Supplementary File 2

*Social Network Analysis*

For the intervention group (N=142), we calculated the proportion of team mates who were also friends. Preliminary analysis showed that a higher friend density within the team did not encourage an increase in average weekly minutes of moderate and vigorous physical activity (MVPA).

**Table S3: Proportion of nominated friends in teams**

| Friends in team | Number of students |
| --- | --- |
| 0 (no nominations) | 5 |
| 0% | 80 |
| 25% | 33 |
| 33% | 8 |
| 50% | 13 |
| 67% | 2 |
| 75% | 1 |

**Figure S1: MVPA (minutes) at baseline and 6 months: relationship with friendship density in team.**


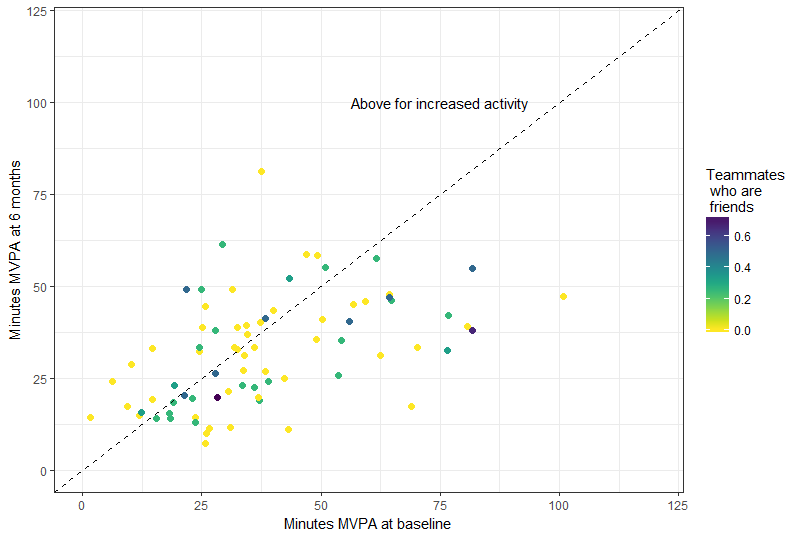


Each point in Figure S1 represents a student in the intervention group, with darker points indicating higher friend density in the assigned team. Participants represented by points above the dashed line increased their minutes of MVPA. Darker points are distributed approximately equally on both sides of the dashed line, showing the lack of association between friend density and change in physical activity.

This interpretation is supported by the distribution of change in physical activity levels by friendship density at Figure S2. The distribution is similar for the participants with no friends in their team as those participants with a higher proportion of friends.

**Figure S2: Change in minutes of MVPA from baseline to 6 months: relationship with friendship density in team.**


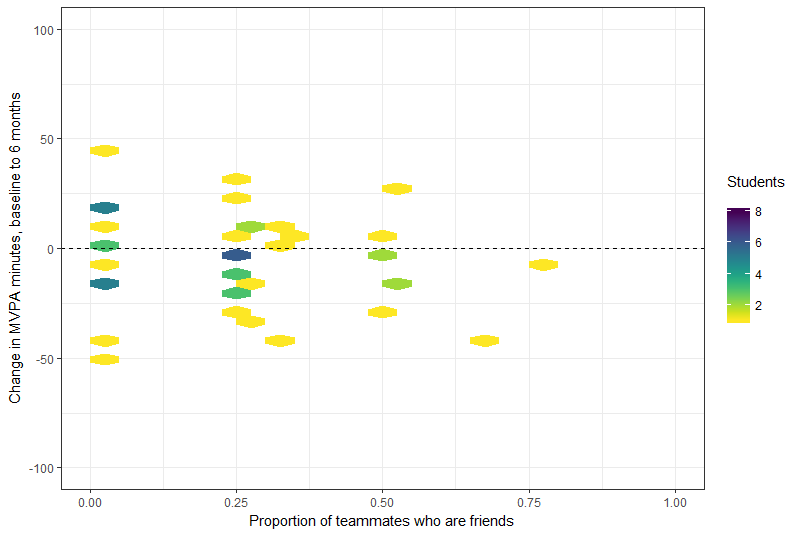

Supplement: Supplementary file 2 — Additional file 2: Table S2. Accelerometer data for participating schools. Table S3. Proportion of nominated friends in teams. Figure S1. MVPA (minutes) at baseline and 6 months: relationship with friendship density in team. Figure S2. Change in minutes of MVPA from baseline to 6 months: relationship with friendship density in team. [file 40814_2019_523_MOESM2_ESM.docx]
